# Patients’ Satisfaction in the Emergency Department: Measurement, Indicators, Factors Influencing Satisfaction, Impact, and Solution

**DOI:** 10.7759/cureus.82392

**Published:** 2025-04-16

**Authors:** Courage Idahor, Bekky E Ben-Igwenyi, Uchechukwu Okonkwo, Oyinkansola Femi-Oyewole, Omo A Ogbeide

**Affiliations:** 1 Emergency Medicine, Barking, Havering and Redbridge University Hospitals NHS Trust, London, GBR; 2 General Practice, St. Helens and Knowsley Teaching Hospitals NHS Trust, London, GBR; 3 General Practice, NES Healthcare UK, London, GBR; 4 General Practice, Chaucer Hospital, Canterbury, GBR

**Keywords:** interpersonal skill, patient-centered care, patient feedback, patient satisfaction metric, quality of healthcare

## Abstract

The emergency department (ED) plays a crucial role in the care of patients, being the first point of contact for patients with the hospital system. Patient satisfaction metrics are crucial indicators of ED care quality, which can significantly impact other hospital departments. Increased patient satisfaction in the ED is associated with better outcomes and fosters trust in the health system. This article explores the key factors influencing patient satisfaction and solutions to enhance patient satisfaction.

## Introduction and background

The emergency department (ED) is usually the first point of contact with the hospital, which gives patients a first impression as they navigate the hospital. Patients’ experience in the ED has become a key differentiator and an important topic of discourse in recent years as the ED records increasing patient attendances with a focus on delivering optimum care [1,2]. In recent times, patient-centered care has progressively been the focus of healthcare transformation efforts by healthcare leaders, and this stresses individualized care and shared decision-making [3,4]. There is evidence from the literature that correlates the experience of patients in the ED with their clinical outcome, hospital profitability, and other healthcare goals, with good overall experience leading to improved patient outcomes and profitability [1,5,6]. There are several factors that impact the experience of patients in the ED. They include but are not limited to patient-ED provider interaction, interpersonal skills, perceived staff attitudes, information dispensation, wait times, communication, cultural aspects of care, patient demographics, pain, staff competence, staff empathy, and compassion [5].

Patient satisfaction metrics are important indicators of emergency care quality. EDs handle 28% of acute care visits in the USA and perhaps even more than in some other countries. At the heart of the hospital operations, the performance of the ED is suitably positioned to influence the perception of other departments in the hospitals [3,7,8]. To improve clinical care and overall patient experience in ED, patient satisfaction metrics need to be explored and workable solutions proffered to address some challenges in ensuring ED meet the needs of patients, enhance their perception of the experience, and exceed their expectations [1]. This opinion article seeks to explore the various factors influencing patient satisfaction, impact, and way forward.

## Review

Effective communication, such as promptly sharing test results, serves as a key indicator of patient satisfaction in the ED. Failure to do this could lead to the patient thinking the healthcare provider does not care about them [3]. In a situation where a patient is not able to speak the same language as the healthcare provider, offering a trained professional interpreter provides a better quality of care and improves patient satisfaction [3]. Moreover, nurses are the backbone of EDs and the “face” of the care that patients receive, so being treated by the nurses with professionalism, courtesy, and respect would make the patient feel cared for. This same service of care should be offered by doctors to patients as it's known that effective doctor-patient communication is critically important in delivering high-quality ED care [3]. Upon discharge, the patient should have understood the possible cause of their health problem and the follow-up care needed [3]. There have been several studies that have established that poor patient experiences during and after discharge could have been avoided through improved communication. Likelihood to recommend is regarded as a key indicator of patient satisfaction, alongside patients' overall hospital rating [3]. Patient satisfaction is a crucial measure of healthcare quality in the ED [9], with numerous influential factors at play. Understanding these factors can guide healthcare providers and hospital administrators in improving patient experience. Among these factors, effective communication [10,11] and positive interpersonal interactions with personnel [12,13] are consistently identified as elements shaping patient satisfaction in the ED.

Satisfaction increases when patients feel there is good communication between them and ED providers [13]. Patients consider communication effective when they receive information on the department’s processes, actual wait times, and unambiguous explanations of their test results, diagnosis, and treatment [10,13,14]. Similarly, quality interpersonal interaction with ED providers strongly predicts patient satisfaction [12,14]. Poor interpersonal exchanges can lead to patient frustration and dissatisfaction [14]. However, fostering positive interpersonal skills such as active listening, open body language, and empathetic statements can significantly improve patient satisfaction [11,15]. In essence, effective communication and good interpersonal interaction make patients feel involved in the decisions regarding their care, thereby enhancing their satisfaction [16]. In addition, there is a correlation between wait times and patient satisfaction [13]. Patient satisfaction correlates more with perceived wait times than actual wait times. Patients care less about actual wait times if they receive timely updates about actual wait times and have good interactions with ED personnel [17,18]. Environmental factors that promote comfort can also influence patient satisfaction. These include parking availability, reduced noise levels, cleanliness, and bed space availability [10]. In addition, timely administration of pain relief and offering hot/cold drinks, blankets, and pillows will increase patient comfort and enhance satisfaction [10,19,20]. Patient factors such as age, social status, ethnicity, and severity of illness have been identified as influencers of patient satisfaction [13,21]. Studies indicate that older patients generally report higher satisfaction than younger patients, and individuals in rural areas often express greater satisfaction than those in urban settings. Moreover, individuals reporting poorer health are less satisfied than their healthier counterparts [21]. In addition, a patient’s perception of a provider based on physical appearance and gender can shape the patient’s experience [21].

Patient satisfaction is also keenly linked to their awareness of their rights in the healthcare setting [22]. This can be achieved through effective information dissemination and communication by healthcare workers. Patients respond positively when they are made to understand their right to autonomy, consent, privacy, and decision-making in the healthcare system. Empowering patients and helping them take ownership of their care increases their confidence in their provider and correlates with increased satisfaction in the healthcare decision-making process [22]. Overcrowding is another factor that affects patient satisfaction in the ED [23]. Overcrowding may be caused by a high volume of patients, reduced bed space resulting in corridor care, and lack of trained healthcare personnel and manpower [23].

While some factors, like environmental comfort and patient characteristics, may be less modifiable, others, such as communication and interpersonal interactions, present opportunities for improvement. Ensuring clear communication regarding processes, wait times, and treatment options, coupled with fostering positive interactions characterized by empathy and active listening, can significantly enhance patient satisfaction. The impact of patient satisfaction in EDs cannot be overemphasized, as it significantly affects both clinical outcomes and service delivery. High satisfaction levels are associated with better patient compliance, reduced rates of readmission, and an overall boost in health outcomes. On the other hand, dissatisfaction can lead to increased complaints, legal actions, and a general lack of faith in the healthcare system and providers. Furthermore, patient satisfaction scores are many times used as a criterion for hospital funding and accreditation, and this subsequently influences how resources are allocated and prioritized within healthcare institutions [24].

Satisfaction in the ED can also affect the morale and retention of healthcare staff. Positive feedback from patients often influences job satisfaction and motivates staff, while negative experiences may contribute to burnout and high turnover rates [24]. Consequently, the implications of patient satisfaction extend beyond the patient-provider interaction, influencing the healthcare ecosystem at large.

Solutions to enhance patient satisfaction in EDs

Optimizing the Triage Processes

Enforcing efficient and effective triage systems can reduce waiting times, thereby making sure that patients receive care based on the severity of their ailments. This not only improves the patient's experience but also optimizes the allocation of resources within the ever-busy ED [25].

Enhancing Communication

Effective communication skills can also improve patient satisfaction. Patients are likely to feel more at ease when clear explanations with regard to the waiting times, diagnosis, management, and treatment plans are provided to them. Transparent communication helps in managing patient expectations and reducing anxiety among patients and their families [26].

Patient-Centered Care Practices

Managing patients holistically can improve patient satisfaction. All aspects of the patient's well-being, including physical comfort, religion, emotional well-being, cultural preferences, and sexual orientation, must be taken into consideration. This could be as simple as being mindful of gender orientations and pronouns to adjusting the physical environment within the department to make it more comfortable for the patients [27].

Implementing Patient Feedback Mechanisms

Regular collation and audit of patient feedback in the department can often provide some degree of insight into patient perception of the care that has been delivered and areas that may need some improvement. Hospitals can use this data to make informed decisions about policy and practice changes, directly addressing the concerns and needs of their patients [28].

Technology Integration

Making use of technological advancements such as mobile apps for updating patients about the wait times and for information dissemination can markedly improve patient satisfaction. These digital tools can provide real-time updates about the progress of the queue and treatment status. They can also possibly reduce anxiety and frustration amongst patients as their expectations are better managed [29].

By focusing on these solutions, EDs can create a more responsive, efficient, and patient-friendly environment, leading to higher satisfaction rates and better health outcomes.

## Conclusions

Patient satisfaction plays an important role in improving overall health outcomes as it fosters trust and open communication between patients and healthcare providers. When patients feel heard and valued, they are more likely to adhere to treatment plans, attend follow-up appointments, and engage in preventive care. This not only improves individual health outcomes but also contributes to more efficient and effective healthcare systems. The ED is often the first point of contact for patients, shaping their general perception of the healthcare system. To improve patient satisfaction in the ED, metrics should be employed to assess patient satisfaction and overall healthcare quality. Some of the solutions to enhance patient satisfaction in the ED include optimising the triage processes, enhancing communication, adopting patient-centered care practices, implementing patient feedback mechanisms, and integrating technology.
